# Disrupted functional connectivity in PD with probable RBD and its cognitive correlates

**DOI:** 10.1038/s41598-021-03751-5

**Published:** 2021-12-21

**Authors:** Javier Oltra, Anna Campabadal, Barbara Segura, Carme Uribe, Maria Jose Marti, Yaroslau Compta, Francesc Valldeoriola, Nuria Bargallo, Alex Iranzo, Carme Junque

**Affiliations:** 1 Medical Psychology Unit, Department of Medicine, Institute of Neurosciences, University of Barcelona, Barcelona, Catalonia Spain; 2 Institute of Biomedical Research August Pi i Sunyer (IDIBAPS), Barcelona, Catalonia Spain; 3 Centro de Investigación Biomédica en Red Enfermedades Neurodegenerativas (CIBERNED), Hospital Clínic de Barcelona, Barcelona, Catalonia Spain; 4 Research Imaging Centre, Campbell Family Mental Health Research Institute, Centre for Addiction and Mental Health (CAMH), University of Toronto, Toronto, ON Canada; 5 Parkinson’s Disease and Movement Disorders Unit, Neurology Service, Hospital Clínic de Barcelona, Institute of Neurosciences, University of Barcelona, Barcelona, Catalonia Spain; 6 Centre de Diagnòstic per la Imatge (CDI), Hospital Clínic de Barcelona, Barcelona, Catalonia Spain; 7 Multidisciplinary Sleep Unit, Neurology Service, Hospital Clínic de Barcelona, University of Barcelona, Barcelona, Catalonia Spain

**Keywords:** Parkinson's disease, Functional magnetic resonance imaging

## Abstract

Recent studies associated rapid eye movement sleep behavior disorder (RBD) in Parkinson’s disease (PD) with severe cognitive impairment and brain atrophy. However, whole-brain functional connectivity has never been explored in this group of PD patients. In this study, whole-brain network-based statistics and graph-theoretical approaches were used to characterize resting-state interregional functional connectivity in PD with probable RBD (PD-pRBD) and its relationship with cognition. Our sample consisted of 30 healthy controls, 32 PD without probable RBD (PD-non pRBD), and 27 PD-pRBD. The PD-pRBD group showed reduced functional connectivity compared with controls mainly involving cingulate areas with temporal, frontal, insular, and thalamic regions (*p* < 0.001). Also, the PD-pRBD group showed reduced functional connectivity between right ventral posterior cingulate and left medial precuneus compared with PD-non pRBD (*p* < 0.05). We found increased normalized characteristic path length in PD-pRBD compared with PD-non pRBD. In the PD-pRBD group, mean connectivity strength from reduced connections correlated with visuoperceptual task and normalized characteristic path length correlated with processing speed and verbal memory tasks. This work demonstrates the existence of disrupted functional connectivity in PD-pRBD, together with abnormal network integrity, that supports its consideration as a severe PD subtype.

## Introduction

Rapid eye movement (REM) sleep behavior disorder (RBD) is a parasomnia characterized by vivid dreams associated with complex movements and dream-enacting behaviors, increased electromyographic activity, and loss of normal muscle atonia during REM sleep^1, 2^. Evidence suggests that isolated RBD is a prodromal symptom of Parkinson’s disease (PD) and other synucleinopathies, with an up to 90% 15-year rate of progression to a defined condition^3^. Furthermore, the prevalence of RBD in PD patients is around 40%^4^. Postmortem studies reveal more diffuse and severe deposition of synuclein in PD patients with RBD^5^.

Structural neuroimaging studies show that the presence of RBD in PD is associated with greater gray matter atrophy by means of voxel-based morphometry^6, 7^ and cortical thickness analysis^7^ in PD patients with video-polysomnographic (vPSG) confirmed RBD diagnosis; as well as by means of deformation-based morphometry^8^, and voxel-based morphometry^9^ in PD patients with questionnaire-based probable RBD status (PD-pRBD). Note that in the case of using questionnaires PD patients can be classified as PD-pRBD or PD without probable RBD (PD-non pRBD). Clinically, a recent meta-analysis shows that PD with vPSG confirmed RBD diagnosis and PD-pRBD are related to disease duration, increased Hoehn and Yahr stage, and higher Movement Disorder Society Unified Parkinson's Disease Rating Scale (MDS-UPDRS) Part III score^10^. Moreover, PD-pRBD is associated with lower cognitive performance^11, 12^, a higher prevalence of mild cognitive impairment^13^, and faster cognitive decline^11^.

Concerning resting-state functional MRI (rs-fMRI) prior works in PD with vPSG confirmed RBD diagnosis found decreased functional connectivity between the pedunculopontine nucleus and the anterior cingulate cortex^14^, decreased amplitude of low-frequency fluctuations in the primary motor and premotor cortices^15^, as well as reduced posterior functional connectivity based on right superior occipital gyrus^16^. Contrary to region-centered approaches, a network-based perspective conceptualizes the brain as a complex network and allows characterizing dynamic interactions between regions through Network-Based Statistics (NBS) and graph-derived metrics^17^. In this context, Li et al.^18^, by graph-derived metrics, found extensive changes of nodal properties in PD-pRBD than PD-non pRBD in comparison with healthy controls in the neocortex and limbic system; as well as enhanced nodal efficiency in the bilateral thalamus and betweenness centrality in the left insula, and reduced betweenness centrality in the right dorsolateral superior frontal gyrus in PD-pRBD compared with PD-non pRBD. The development of the threshold-free network-based statistics (TFNBS) method^19^, which, unlike NBS, does not require the a priori definition of a component-defining threshold and generates edge-wise significant values, has been proposed as a step forward. Recently, this approach revealed in isolated RBD a disruption of posterior functional connectivity^20^. Nevertheless, as far as we know, there is no previous literature in PD-pRBD studying rs-fMRI interregional functional connectivity through network-based statistics. Our main aim is to characterize dysfunction of brain connectivity in PD-pRBD using TFNBS whole-brain and graph theory analyses and to investigate its possible relation with cognitive dysfunctions. We hypothesize that PD patients with probable RBD will show a reduction in brain functional connectivity compared with healthy controls and PD patients without probable RBD and the reduction will be associated with cognitive impairment.

## Methods

### Participants

Seventy-one PD patients were recruited from the Parkinson’s Disease and Movement Disorders Unit (Hospital Clínic de Barcelona, Barcelona, Spain); and 69 voluntary healthy controls recruited from the Institut Català de l'Envelliment (Universitat Autònoma de Barcelona, Barcelona, Catalonia, Spain) and patients’ relatives. The patient’s inclusion criteria were: (a) attaining UK PD Society Brain Bank diagnostic criteria for PD and (b) no treatment with deep-brain stimulation. Exclusion criteria were: (a) mild cognitive impairment (MCI) for healthy controls, (b) PD age of onset less than 40 years; (c) age less than 50 years; (d) severe comorbidity due to psychiatric or neurological conditions; (e) score below 25 obtained in Mini-Mental State Examination (MMSE) for healthy controls; (f) claustrophobia; (g) pathological MRI findings apart from mild white matter hyperintensities in the fluid-attenuated inversion recovery (FLAIR) acquisition; (h) MRI artifacts; (i) absence of fMRI resting-state acquisition; (j) fMRI head motion parameter of mean interframe head motion at ≥ 0.3 mm translation or 0.3° rotation; (k) fMRI head motion parameter of maximum interframe head motion at ≥ 1 mm translation or 1° rotation; (l) no response to Innsbruck RBD Inventory; (m) probable RBD (pRBD) condition based on Innsbruck RBD Inventory for healthty controls. Inclusion criteria and exclusion criteria (b) to (g) were also used in Uribe et al. 2016^21^.

After applying the criteria, we selected 59 PD patients and 30 healthy controls. The excluded PD participants were: 1 because of young-onset, 1 for age < 50 years, 1 for young-onset and age < 50 years, 1 for vascular parkinsonism lookalike condition, 1 for claustrophobia, 2 for absence of fMRI, 5 for no Innsbruck RBD Inventory response. From healthy controls participants, were excluded: 4 for age < 50 years, 3 for psychiatric comorbidity, 8 for MCI condition, 1 for MMSE score below 25, 1 for MRI artifact, 1 for MCI condition and MRI artifact, 3 for fMRI head motion parameters, 5 for no Innsbruck RBD Inventory response, 13 for pRBD condition.

PD patients were classified in PD-pRBD (n = 27) and PD-non pRBD (n = 32) following the 5-item test Innsbruck REM Sleep Behavior Disorder Inventory, with a 0.25 cutoff (number of positive symptoms/number of answered questions)^22^.

The study had the approval of the Ethics Committee of the University of Barcelona (IRB00003099) and Hospital Clínic (HCB/2014/0224). All participants provided written informed consent after a full explanation of the procedures involved. It was performed in accordance with relevant regulations and guidelines.

### Neuropsychological and clinical assessment

All subjects underwent a neuropsychological battery including Digit Span Forward and Backward (WAIS), phonemic fluency (letter ‘p’), semantic fluency (animals), Stroop Color and Word Test, Trail Making Test (TMT), Symbol Digits Modalities Test-Oral version (SDMT), Rey Auditory Verbal Learning Test (RAVLT), Benton Judgment of Line Orientation (JLO), Benton Visual Form Discrimination (BVFD), Benton Facial Recognition Test short form (27-item, BFRT), Boston Naming Test (BNT). The presence of mild cognitive impairment (MCI) was established as in a previous study based on z scores adjusted for age, sex, and education extracted by a multiple regression analysis^23^ performed in a healthy control reference group^24^.

Clinical evaluation included motor symptoms assessed with the MDS-UPDRS Part III, disease severity with Hoehn and Yahr scale, global cognition with the MMSE, and olfactory function using the University of Pennsylvania Smell Identification Test (UPSIT-40)^25^.

l-dopa equivalent daily dose (LEDD)^26^ was calculated for standardization purposes by the different doses of antiparkinsonian drugs that the PD patients took.

### MRI acquisition

MRI acquisition with a 3 T scanner (MAGNETOM Trio, Siemens, Germany). The scanning protocol included: (a) high-resolution 3-dimensional T1-weighted images acquired in the sagittal plane repetition time = 2300 ms, echo time = 2.98 ms, inversion time = 900 ms, 240 slices, field-of-view = 256 mm; 1 mm isotropic voxel); (b) axial FLAIR sequence (repetition time = 9000 ms, echo time = 96 ms); and (c) resting-state 10-min-long functional gradient-echo echo-planar imaging sequence (240 T2^∗^ weighted images, repetition time = 2.5 s, echo time = 28 ms, flip angle = 80°, slice thickness = 3 mm, field-of-view = 240 mm). Subjects were instructed to keep their eyes closed, not to fall asleep, and not to think anything in particular. The same acquisition protocol was used in Campabadal et al. 2020^20^.

### MRI preprocessing

Main functional image preprocessing, using AFNI tools, described in Campabadal et al. 2020^20^ included “discarding the first five volumes to allow magnetization stabilization, despiking, motion correction, grand-mean scaling, linear detrending, and temporal filtering (maintaining frequencies above 0.01 Hz)”. Moreover, the preprocessing included an Independent Component Analysis (ICA-AROMA)^27^ based strategy for Automatic Removal of Motion Artifacts^20^, along with a quality control based on correlations between framewise head displacement and overall signal variation^28^.

### Characterization of brain functional connectivity and network properties

To test for intergroup differences in interregional connectivity, we applied threshold-free network-based statistics (TFNBS)^19^. This approach allows performing statistical inference on brain graphs through network-based statistics^29^ and threshold-free cluster enhancement^30^. One of the main characteristics of TFNBS is the estimation of edge-wise significance values, which is useful for the selection of relevant connectivity features. The 246 regions defined in the Brainnetome Atlas (https://atlas.brainnetome.org/bnatlas.html) were used for the characterization of global functional connectivity (for a detailed list of the used nodes see Supplementary Methods 1)^31^.

Complementary, a graph theory implementation was applied to describe the network topology through its global (whole-brain) and local (nodal) properties^32, 33^. The extraction of the global and local parameters, using Brain Connectivity Toolbox (BCT), included: clustering coefficient, node degree, small-worldness, path length, efficiency, and betweenness centrality. For detailed definitions and calculations of these graph metrics, see Rubinov and Sporns^33^. Computation used nine different density thresholds (maintaining the 5% to 25% strongest edges, at intervals of 2.5%), followed by a reporting results criterion of significance in more than 75% of the thresholds.

### Statistical analyses

Group differences were conducted in demographic, neuropsychological and clinical variables using IBM SPSS Statistics 27.0.0 (2020; Armonk, NY: IBM Corp) by analysis of variance (ANOVA) or analysis of covariance (ANCOVA) followed by Bonferroni or Games-Howell post hoc tests, or Kruskal–Wallis H and Mann–Whitney U tests as appropriate. Differences in categorical measures were analyzed by Pearson's chi-squared. To perform correlation analyses between neuroimaging and neuropsychological variables, the Pearson and Spearman correlation coefficients were applied. The statistical significance threshold was set at *p* < 0.05.

Between groups differences in connectivity measures were tested with the general linear model using in-house MATLAB scripts. Statistical significance was established using Monte Carlo simulations with 5,000 permutations. Two-tailed p-values were calculated as the proportion of values in the null distribution more extreme than those observed in the actual model.

## Results

### Sociodemographic, clinical, and neuropsychological data

Table 1 shows the sociodemographic and clinical characteristics of participants. There were no significant differences between groups in age and education. However, differences between healthy control and PD groups were observed for sex, subsequent analyses that included both groups were controlled by this variable. The PD groups were similar on age of onset, LEDD, and disease severity. PD-pRBD showed slightly longer disease duration (not reaching statistical significance *p* = 0.055), even so, this variable was controlled in subsequent comparison between PD groups. Both PD groups differed from controls in olfactory function.

**Table 1:** Sociodemographic, clinical, and head motion comparisons among PD-non pRBD, PD-pRBD and HC.

	PD-non pRBD (n = 32)	PD-pRBD (n = 27)	HC (n = 30)	Test stat	p-value
**Sociodemographic and clinical data**					
Sex, male, n (%)	21 (65.6)	23 (85.2)	13 (43.3)	10.863^a^	0.004^f,g^
Age, y, mean (SD)	64.5 (9.9)	68.8 (9.2)	67.5 (7.7)	1.827^b^	0.167
Education, y, mean (SD)	12.8 (5.3)	11.4 (5.4)	11.4 (4.0)	1.255^c^	0.534
Age of onset, y, mean (SD)	58.2 (10.5)	59.2 (9.6)	NA	0.378^d^	0.707
Disease duration, y, mean (SD)	6.3 (3.6)	8.6 (5.0)	NA	1.977^d^	0.055
LEDD, mean (SD)	516.6 (277.4)	707.8 (469.2)	NA	336.5^e^	0.146
MDS-UPDRS-Part III, mean (SD)	15.4 (10.2)	17.1 (8.1)	NA	314.5^e^	0.228
H&Y stage, n, 1/2/2.5/3/4	6/17/1/6/0	2/11/0/10/1	NA	5.689^a^	0.224
UPSIT, mean (SD)	18.9 (6.8)	16.8 (6.8)	29.2 (4.3)	39.319^c^	< 0.001^f,g^
RBD-I, symptoms/answers, mean (SD)	0.08 (0.09)	0.56 (0.14)	0.05 (0.07)	59.453	< 0.001^g,h^
**Mean interframe head motion**					
Rotation, degrees, mean (SD)	0.051 (0.033)	0.051 (0.030)	0.039 (0.020)	2.215^c^	0.330
Translation, mm, mean (SD)	0.100 (0.057)	0.095 (0.043)	0.124 (0.058)	2.523^b^	0.086
**Maximum interframe head motion**					
Rotation, degrees, mean (SD)	0.308 (0.215)	0.327 (0.200)	0.267 (0.165)	0.840^c^	0.657
Translation, mm, mean (SD)	0.377 (0.190)	0.481 (0.211)	0.502 (0.170)	7.164^c^	0.028^f^

*PD-non pRBD* Parkinson’s disease patients without probable REM sleep behavior disorder, *PD-pRBD* Parkinson’s disease patients with probable REM sleep behavior disorder, *HC* healthy controls, *y* years, *LEDD* levodopa equivalent daily doses, *MDS-UPDRS* Movement Disorder Society Unified Parkinson's Disease Rating Scale, *H&Y *Hoehn and Yahr scale, *UPSIT* University of Pennsylvania Smell Identification Test, *RBD-I* Innsbruck REM Sleep Behavior Disorder Inventory.

^a^Chi-squared test.

^b^Analysis of variance (ANOVA) test followed by Bonferroni post-hoc test.

^c^Kruskal–Wallis H test followed by Mann–Whitney U test.

^d^t-test.

^e^Mann–Whitney U test.

^f^Post-hoc differences between PD-non pRBD and HC (*p* < 0.05).

^g^Post-hoc differences between PD-pRBD and HC (*p* < 0.05).

^h^Post-hoc differences between PD-non pRBD and PD-pRBD (*p* < 0.05).

Table 2 describes neuropsychological results by group. Inter-group comparisons showed PD-pRBD patients had lower performance than PD-non pRBD and healthy controls in Stroop Word and Word-Color and TMT-A. PD-pRBD patients also had significantly lower scores than healthy controls in Stroop Color, semantic fluency, RAVLT Total, JLO, SDMT, TMT B and B minus A, as well as BFRT-short. PD-pRBD differed from PD-non pRBD in Stroop Word, Color and Word-Color when controlling by disease duration (Supplementary Table 1). There were no between PD groups differences in MCI distribution (Supplementary Table 2). To facilize the interpretation of neuropsychological data we include the descriptive statistics in z-scores calculated based on an healthy control reference group means and standard deviations^24^ in Supplementary Table 3.

**Table 2:** Neuropsychological performance of PD-non pRBD, PD-pRBD and HC.

Test	PD-non pRBD	PD-pRBD	HC	Test stat (p-value)
**MMSE**	29.06 (1.24)	28.07 (2.93)	29.13 (0.94)	3.265 (0.043)
**Digit span**				
Forward	5.38 (1.21)	5.44 (1.28)	5.57 (1.33)	0.675 (0.512)
Backward	3.97 (1.00)	4.11 (1.34)	4.10 (0.92)	0.298 (0.743)
Forward minus backward	1.41 (1.41)	1.33 (1.21)	1.47 (0.90)	0.210 (0.811)
**Phonetic fluency "p"**	15.47 (6.04)	6.04 (5.29)	15.77 (5.96)	1.368 (0.260)
**Semantic fluency "animals"**	16.94 (6.51)	15.07 (7.73)	20.57 (4.08)	6.136 (0.003)^a^
**Stroop**				
Word	89.74 (17.42)	78.26 (23.32)	97.07 (15.39)	8.309 (0.001)^a,b^
Color	56.32 (13.83)	47.92 (16.25)	64.30 (9.90)	7.431 (0.001)^a^
Word–Color	34.55 (11.80)	26.96 (13.42)	37.03 (9.09)	5.918 (0.004)^a,b^
**TMT**				
A	51.63 (35.24)	85.11 (98.20)	38.93 (10.85)	6.022 (0.004)^a,b^
B	146.43 (143.93)	189.50 (215.69)	95.87 (46.23)	3.394 (0.039)^a^
B minus A	96.07 (115.84)	138.73 (196.38)	56.93 (39.83)	3.038 (0.054)^a^
**SDMT**	41.91 (14.79)	37.37 (18.38)	47.50 (8.47)	3.792 (0.026)^a^
**RAVLT**				
Total	44.06 (10.38)	38.96 (11.38)	46.00 (6.21)	3.246 (0.044)^a^
Recall	8.22 (3.66)	7.56 (3.46)	9.43 (2.06)	2.088 (0.130)
True recognition	13.22 (2.76)	13.44 (1.72)	14.13 (1.31)	1.591 (0.210)
JLO	23.81 (5.72)	22.00 (6.87)	25.20 (3.40)	5.537 (0.005)^a^
BVFD	29.44 (2.61)	27.93 (4.11)	29.17 (2.35)	2.534 (0.085)
BFRT-short	21.66 (2.62)	20.96 (2.89)	23.17 (1.97)	5.631 (0.005)^a^
BNT	13.56 (1.22)	13.48 (1.01)	13.67 (0.88)	0.970 (0.383)

Data are presented as mean (SD) of raw scores. Analyses of covariance (ANCOVA) with sex as covariate, followed by Bonferroni post-hoc tests.

*PD-non pRBD* Parkinson’s disease patients without probable REM sleep behavior disorder, *PD-pRBD* Parkinson’s disease patients with probable REM sleep behavior disorder, *HC* healthy controls, *MMSE* Mini-Mental State Examination, *TMT *Trail Making Test, *SDMT* Symbol Digit Modalities Test,  *RAVLT *Rey Auditory Verbal Learning Test, *JLO* Benton Judgment of Line Orientation, *BVFD* Benton Visual Form Discrimination,* BFRT* Benton Facial Recognition Test, *BNT *Boston Naming Test.

^a^Post-hoc differences between PD-pRBD and HC (*p* < 0.05).

^b^Post-hoc differences between PD-pRBD and PD-non pRBD (*p* < 0.05).

### Functional connectivity and network graph metrics

Significant difference between healthy controls and PD-non pRBD group was found for maximum translation (Table 1), hence it was introduced as a covariate in all subsequent analyses that included these two groups.

**Figure 1:**
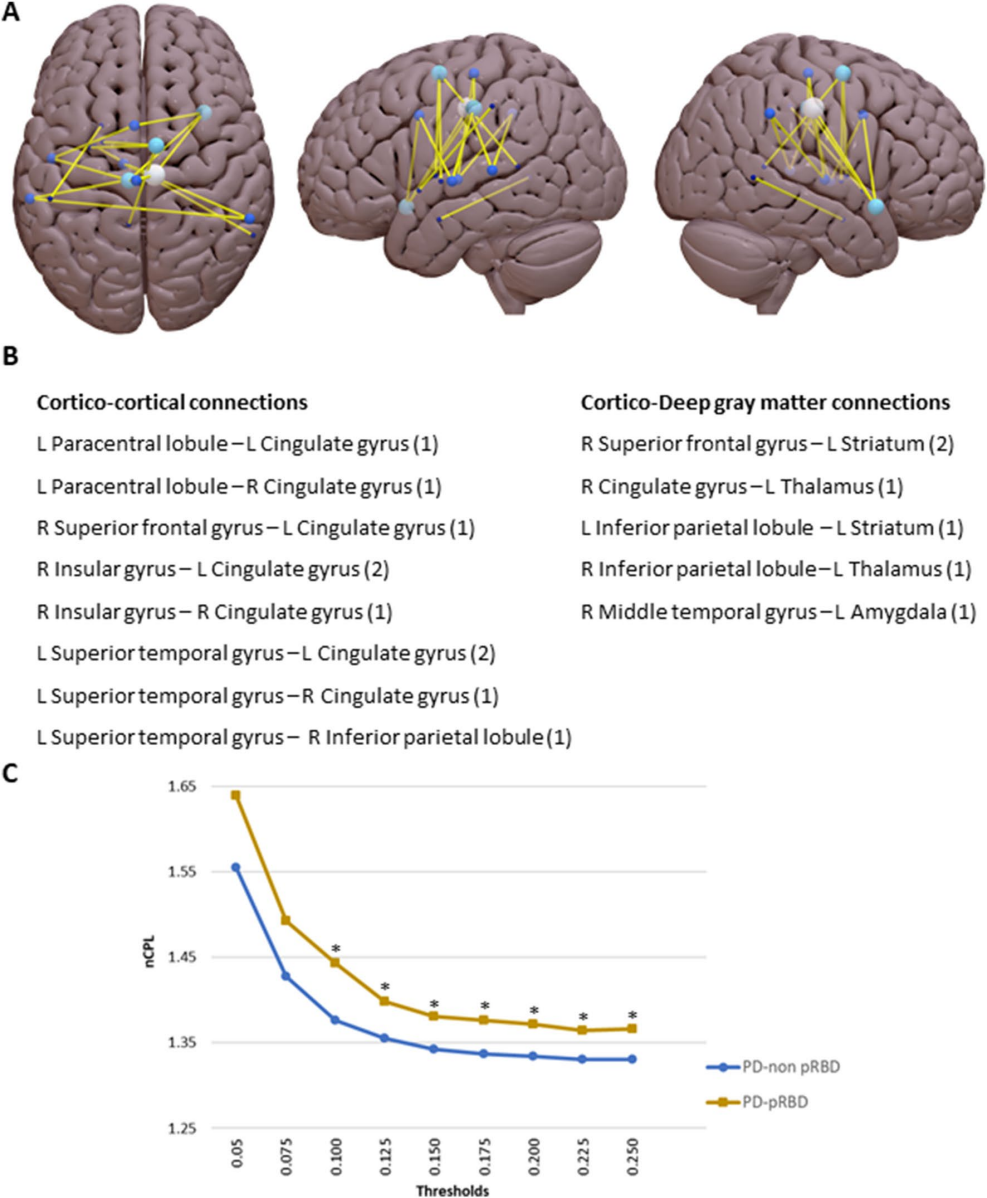
**(A)** Schematic representation of the reduced functional connectivity strength in Parkinson’s disease patients with probable REM sleep behavior disorder (PD-pRBD) compared with healthy controls (HC) in the whole-brain analysis consisting of sixteen edges found to be significantly different between groups. Lighter colors represent nodes connected to a greater number of altered connections. Comparison using threshold-free network-based statistics (*p* < 0.001, FWE corrected) with sex and maximum translation as covariate. **(B)** Summary of the altered edges in PD-pRBD represented in panel A classified in cortico-cortical and cortico-deep gray matter connections. The altered edges are classified in types according to the structures of the nodes involved. To see a more detailed list with the specific edges and their corresponding node pairs see Supplementary Table 4. In parentheses the number of connections of corresponding to each type, e.g. in “R Insular gyrus–L Cingulate gyrus (2)” indicates two altered connections of this type. Regions were defined based on the Brainnetome Atlas (see Supplementary Methods 1). **(C)** Shows the normalized characteristic path length (nCPL) increment in PD-pRBD patients compared with Parkinson’s disease patients without probable REM sleep behavior disorder (PD-non pRBD). Normalized characteristic path length (vertical axis) as a function of sparsity thresholds (horizontal axis) for PD-pRBD and PD-non pRBD. (*) indicate significant differences between PD-non pRBD and PD-pRBD. Brain plots were created with Surf Ice (https://www.nitrc.org/projects/surfice/). *L *left, *R* right.

Whole-brain functional connectivity analysis showed that PD-pRBD had 16 connections with significantly reduced functional connectivity strength when compared with healthy controls (*p* < 0.001, FWE corrected; Fig. 1a,b and Supplementary Table 4 for a detailed list of the altered connections). From the 16 connections, 10 (62.5%) were found to be cortico-cortical edges and 6 (37.5%) were cortico-deep gray matter edges (Supplementary Table 5). When comparing PD groups controlling by disease duration, the PD-pRBD group had significantly reduced functional connectivity strength between the right ventral posterior cingulate (Brodmann area 23) and the left medial precuneus (pEM, Brodmann area 5) that correspond to CG_R_7_4 and Pcun_L_4_2 labels of the Brainnetome Atlas respectively (*p* < 0.05, FWE corrected).

Intergroup difference in global graph parameters showed increased normalized characteristic path length in PD-pRBD patients compared with PD-non pRBD in 7 out of 9 thresholds controlling by disease duration (Fig. 1c).

Additionally, we explored the potential influence of cognitive impairment in functional connectivity and network graph metrics. We performed a whole-brain functional connectivity analysis with the four resulting groups (PD-non pRBD-non MCI = 19, PD-non pRBD-MCI = 13, PD-pRBD-non MCI = 13, PD-pRBD-MCI = 14) and did not find statistical significant differences (*p* < 0.05, FWE corrected). Further, we found increased normalized characteristic path length in PD-pRBD-MCI patients compared with PD-non pRBD-MCI in 9 out of 9 thresholds after applying network graph metrics analyses with those four groups (Supplementary Fig. 1).

### Correlation of cognitive measures with functional connectivity and network graph metrics

**Figure 2:**
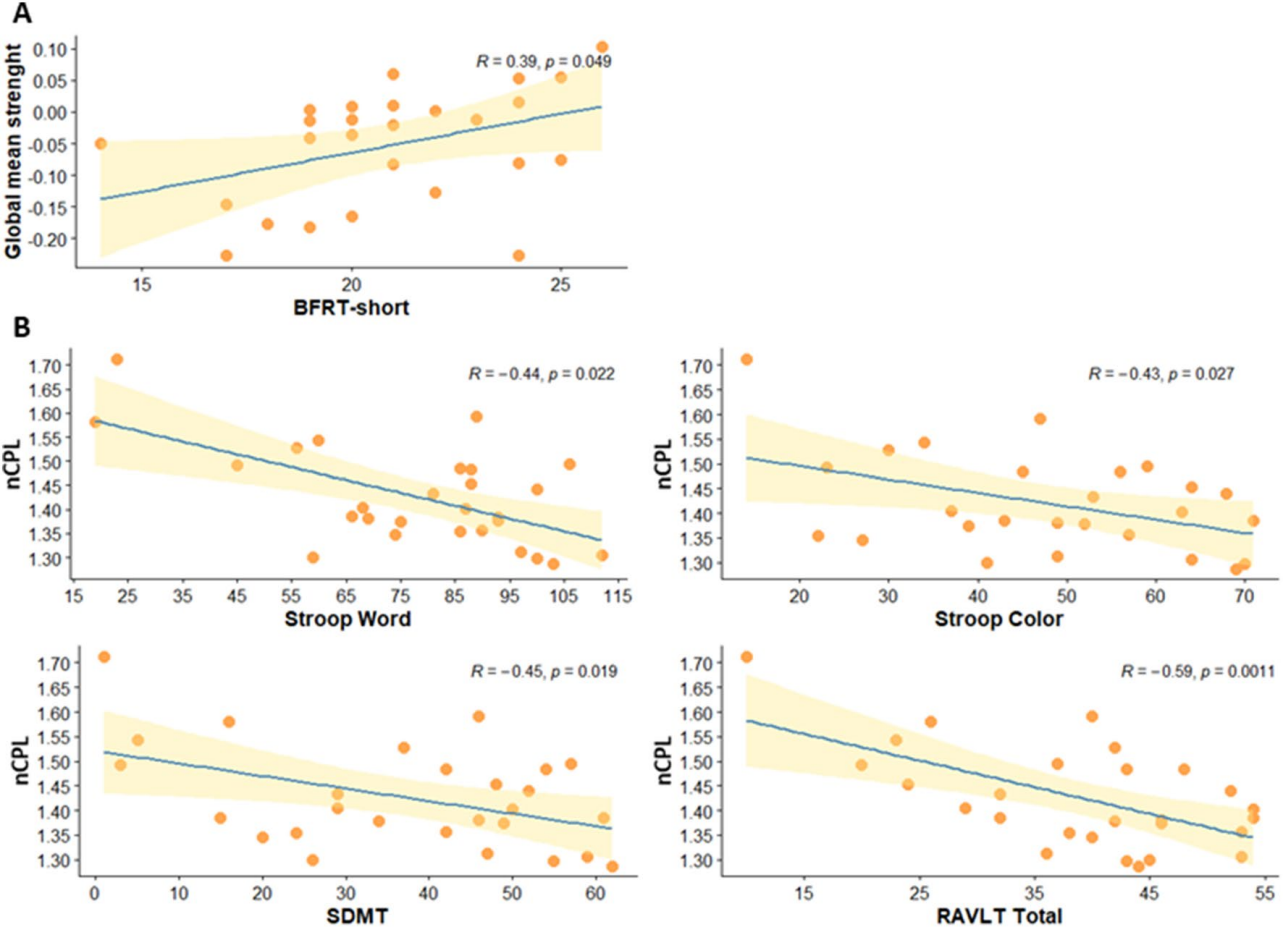
**(A)** Significant correlation between the global mean strength and the BFRT-short (Benton Facial Recognition Test short form) in PD-pRBD group. **(B)** Significant correlations between normalized characteristic path length (nCPL) and Stroop Word, Stroop Color, Symbol Digits Modalities Test-Oral version (SDMT) and Rey Auditory Verbal Learning Test (RAVLT) total in PD-pRBD group. Left to right, top to bottom. Shaded area represents 95% confidence interval.

The global mean strength of the 16 edges was correlated with the neuropsychological measures with significantly lower performance in PD-pRBD compared with the other two groups. Positive correlations were found with BFRT-short (Fig. 2a).

In the PD-pRBD group the normalized characteristic path length correlated with scores in the Stroop Word and Color, SDMT and RAVLT Total (Fig. 2b).

No significant correlations between cognitive measures and functional connectivity measures were found in the PD-non pRBD and healthy control groups.

## Discussion

This is the first work investigating resting-state interregional functional connectivity through whole-brain network-based statistics in PD-pRBD patients. Our results suggest that PD-pRBD patients had reduced resting-state functional connectivity and increased normalized characteristic path length in comparison with healthy controls. PD-pRBD patients showed posterior connectivity disruption compared with PD-non pRBD patients. Moreover, functional abnormalities were associated with cognitive impairment only in the PD-pRBD group.

The whole-brain analyses revealed an extended reduced functional connectivity in PD-pRBD patients compared with healthy controls, mainly involving posterior cingulate areas, and their connections with temporal, frontal, insular and thalamic regions. Besides, we found reduced connectivity between the left superior temporal and the right parietal cortex. Concerning cortico-deep gray matter connection, we also identified a significant decrease in fronto-striatal, parietal-striatal, parietal-thalamic regions, as well as between amygdala and posterior middle temporal cortex. Overall, our results supported the existence of an abnormal connectivity pattern in PD-pRBD patients that mainly included cortical paralimbic connections.

Our findings evidenced a relevant cingulate cortex implication in the pattern shown by PD-pRBD. Previous work also found cingulate cortex abnormalities in a resting-state study with reduced connectivity between the anterior cingulate cortex and the pedunculopontine nucleus, as regions from the arousal network. The authors of that work suggested that decreased connectivity in the arousal network would be related to alertness regulation in PD patients with vPSG RBD confirmed diagnosis^14^. Additionally, we found reduced connectivity between motor and premotor regions and basal ganglia in PD-pRBD compared with healthy controls. In this line, functional abnormalities have been reported previously in motor fronto-striatal circuitry in PD with RBD diagnosed by vPSG using Amplitude of Low-Frequency Fluctuations (ALFF). These results suggested that RBD pathophysiology involves not only midbrain dysfunction but also cortico-subcortical altered connectivity^15^. Of interest, previous evidence pointed to increased REM sleep without atonia in PD with freezing of gait^34^ and subliminal gait initiation deficits in isolated RBD^35^. Future research could explore the relation between both symptoms, freezing of gait and RBD, using functional connectivity approaches.

Our study used a complex approach that allows characterizing the whole brain functional connectivity. Regarding between PD groups comparison, we evidenced disrupted posterior functional connectivity in PD-pRBD between the right ventral area of the cingulate and the left medial area of the precuneus. In this context, a recent work identified reduced posterior functional connectivity in PD with RBD confirmed by vPSG compared with PD without RBD. However, this study used a seed-to-whole brain approach based on a priori region of interest located at the right superior occipital gyrus^16^. It is noteworthy that our group identified disrupted posterior connectivity in isolated RBD applying the same whole-brain methodological approach used in the current study^20^. Altogether might reflect that this regional pattern would be associated with RBD condition in the synucleinopathies spectrum.

On the other hand, our graph analyses reported increased normalized characteristic path length in the PD-pRBD group compared with PD-non pRBD. This finding might reflect global integration and efficiency abnormalities in the network^33^. The unique previous research performed in PD-pRBD reported enhanced nodal efficiency in the thalami and betweenness centrality in the right dorsolateral superior frontal gyrus in PD-pRBD compared with healthy controls. Nevertheless, they did not find differences in global graph metrics between groups^18^. Although an early work reported an incremented normalized characteristic path length in PD patients compared with healthy controls^36^. Further studies showed that global integration seems preserved in early-stage drug-naïve PD patients^37^, but altered in severe disease phenotypes with early-stage PD-MCI^38^. These findings are of great interest considering that pRBD condition is associated with worse cognitive prognosis^11^. In our study, the MCI condition by itself did not explain the obtained between PD groups results from TFNBS and graph metrics. In additional analyses, the found difference in normalized characteristic path length appeared between PD-pRBD-MCI and PD-non pRBD-MCI groups replicated the main result and may indicated that differences could be aggravated between PD-MCI subgroups.

In this context, despite we did not find differences in MCI diagnosis between groups, the PD-pRBD group showed a widespread impairment in the neuropsychological battery with lower performance in mental processing speed, verbal fluency, verbal memory, visuospatial (VS) and visuoperceptual (VP) tasks. This result is in line with previous works in PD-pRBD^11, 12^. Furthermore, we found a significant correlation between the mean functional connectivity strength and a visuoperceptual task in PD-pRBD, as well as significant correlations between the normalized characteristic path length and measures of mental processing speed and verbal learning in PD-pRBD. The role of altered brain functional connectivity in cognitive impairment in PD-pRBD is congruent since an incremented path length implies a less efficient transfer of information due to the altered integrity of the network^39, 40^. Our result concords well with a previous work of our group with isolated RBD patients, finding a correlation between mental processing speed domain and temporoparietal connectivity disruption^20^. It is interesting to note that the mental processing speed domain is assessed with the SDMT and Stroop Color in both studies. It may be assumed that SDMT and Stroop Color test require not only mental processing speed, but also the integration of attention, VS, and VP functions. So, this may be the reason why they are more sensitive when it comes to reflecting brain dysfunctions in patients with RBD. In summary, PD-pRBD patients showed worse cognitive profile and functional connectivity abnormalities suggesting an association between pRBD and severe phenotype.

The main limitation of the present study is the absence of polysomnography-based RBD diagnosis, which is the gold standard for diagnosing RBD. However, the Innsbruck REM sleep behavior disorder inventory had good psychometric properties in PD population, a sensitivity of 0.91, and a specificity of 0.86 (AUC = 0.89)^22^ and has been frequently used to characterize probable RBD in PD in previous studies^41, 42^.

In summary, in this study, we demonstrate the existence of abnormal network integrity and disrupted functional connectivity in PD-pRBD. Furthermore, we found evidence that reduced connectivity was associated with impaired visuoperceptual functions; as well as abnormal functional integrity was associated with lower performance in verbal learning and mental processing speed. Our results underpin the presence of pRBD as a condition related to severe phenotype in PD.

## Supplementary Information

Supplementary Information.

## Data Availability

The data that support the findings of this study are available from the corresponding author upon reasonable request.
