# Supplementary material for: Disrupted functional connectivity in PD with probable RBD and its cognitive correlates
Source: Sci Rep. 2021 Dec 21;11:24351. doi: 10.1038/s41598-021-03751-5 (PMC8692356; doi:10.1038/s41598-021-03751-5)
Supplement: Supplementary file 1 — Supplementary Information. [file 41598_2021_3751_MOESM1_ESM.docx]

**Disrupted functional connectivity in PD with probable RBD and its cognitive correlates**

Javier Oltra^1, 2^, Anna Campabadal^1, 2^, Barbara Segura^1, 2, 3*^, Carme Uribe^1, 2, 4^, Maria Jose Marti^2, 3, 5^, Yaroslau Compta^2, 3, 5^, Francesc Valldeoriola^2, 3, 5^, Nuria Bargallo^2, 6^, Alex Iranzo^2, 3, 7^, Carme Junque^1, 2, 3^

^1^Medical Psychology Unit, Department of Medicine, Institute of Neurosciences, University of Barcelona, Barcelona, Catalonia, Spain

^2^ Institute of Biomedical Research August Pi i Sunyer (IDIBAPS), Barcelona, Catalonia, Spain

^3^Centro de Investigación Biomédica en Red Enfermedades Neurodegenerativas (CIBERNED), Hospital Clínic de Barcelona, Barcelona, Catalonia, Spain

^4^Research Imaging Centre, Campbell Family Mental Health Research Institute, Centre for Addiction and Mental Health (CAMH), University of Toronto, Toronto, Ontario, Canada

^5^ Parkinson’s Disease and Movement Disorders Unit, Neurology Service, Hospital Clínic de Barcelona, Institute of Neurosciences, University of Barcelona, Barcelona, Catalonia, Spain

^6^Centre de Diagnòstic per la Imatge, Hospital Clínic, Barcelona, Catalonia, Spain

^7^Multidisciplinary Sleep Unit, Neurology Service, Hospital Clínic de Barcelona, University of Barcelona, Barcelona, Catalonia, Spain

*Corresponding author. Dr Barbara Segura. Medical Psychology Unit, Department of Medicine, University of Barcelona, Casanova 143, 08036, Barcelona, Catalonia, Spain.  phone [+34] 934039297 // 93 4034446 Fax: [+34] 93 4035294. E-mail addres: bsegura@ub.edu (B.Segura).

E-mail addresses: joltra@ub.edu (J.Oltra),  anna.campabadal@ub.edu (A. Campabadal), bsegura@ub.edu (B. Segura),  carme.uribe@ub.edu (C.Uribe), mjmarti@clinic.cat (M.J. Marti), ycompta@clinic.cat (Y. Compta), fvallde@clinic.cat (F. Valldeoriola), bargallo@clinic.cat (N. Bargallo), [airanzo@clinic.cat](mailto:airanzo@clinic.cat) (A.Iranzo), cjunque@ub.edu (C. Junque).

**Supplementary Material**

**Supplementary Table 1**

Neuropsychological performance of PD-non pRBD and PD-pRBD.

| *Test* | | **PD-non pRBD** | **PD-pRBD** | **Test stat (p-value)** |
| --- | --- | --- | --- | --- |
| MMSE | | 29.06 (1.24) | 28.07 (2.93) | 3.065 (0.086) |
| Digit Span | |  |  |  |
|  | Forward | 5.38 (1.21) | 5.44 (1.28) | 0.212 (0.647) |
|  | Backward | 3.97 (1.00) | 4.11 (1.34) | 0.149 (0.701) |
|  | Forward minus Backward | 1.41 (1.41) | 1.33 (1.21) | 0.008 (0.928) |
| Phonetic fluency "p" | | 15.47 (6.04) | 6.04 (5.29) | 2.815 (0.099) |
| Semantic fluency "Animals" | | 16.94 (6.51) | 15.07 (7.73) | 1.010 (0.319) |
| Stroop | |  |  |  |
|  | Word | 89.74 (17.42) | 78.26 (23.32) | 5.170 (0.027) |
|  | Color | 56.32 (13.83) | 47.92 (16.25) | 4.463 (0.039) |
|  | Word-Color | 34.55 (11.80) | 26.96 (13.42) | 4.711 (0.035) |
| TMT | |  |  |  |
|  | A | 51.63 (35.24) | 85.11 (98.20) | 3.380 (0.071) |
|  | B | 146.43 (143.93) | 189.50 (215.69) | 0.172 (0.680) |
|  | B minus A | 96.07 (115.84) | 138.73 (196.38) | 0.236 (0.629) |
| SDMT | | 41.91 (14.79) | 37.37 (18.38) | 0.633 (0.430) |
| RAVLT | |  |  |  |
|  | Total | 44.06 (10.38) | 38.96 (11.38) | 3.459 (0.068) |
|  | Recall | 8.22 (3.66) | 7.56 (3.46) | 0.698 (0.407) |
|  | True recognition | 13.22 (2.76) | 13.44 (1.72) | 0.034 (0.853) |
| JLO | | 23.81 (5.72) | 22.00 (6.87) | 1.064 (0.307) |
| BVFD | | 29.44 (2.61) | 27.93 (4.11) | 1.382 (0.245) |
| BFRT-short | | 21.66 (2.62) | 20.96 (2.89) | 0.088 (0.768) |
| BNT | | 13.56 (1.22) | 13.48 (1.01) | 0.128 (0.722) |

Data are presented as mean (SD) of raw scores. Analyses of covariance (ANCOVA) with disease duration as covariate, followed by Bonferroni post-hoc tests.

Abbreviations:  PD-non pRBD = Parkinson’s disease patients without probable REM sleep behavior disorder; PD-pRBD = Parkinson’s disease patients with probable REM sleep behavior disorder; MMSE = Mini-Mental State Examination; TMT = Trail Making Test; SDMT = Symbol Digit Modalities Test; RAVLT = Rey Auditory Verbal Learning Test; JLO = Benton Judgment of Line Orientation; BVFD = Benton Visual Form Discrimination; BFRT = Benton Facial Recognition Test; BNT = Boston Naming Test.

**Supplementary Table 2**

Mild cognitive impairment distribution of the PD groups

|  | **PD-non pRBD** | **PD-pRBD** | **Test stat (p-value)** |
| --- | --- | --- | --- |
| MCI, n (%) | 13 (40.6%) | 14 (51.9%) | 0.733 (0.388) |

Pearson's chi-squared was used.

Abbreviations: MCI = mild cognitive impairment; PD-non pRBD = Parkinson’s disease patients without probable REM sleep behavior disorder; PD-pRBD = Parkinson’s disease patients with probable REM sleep behavior disorder.

**Supplementary Table 3**

| *Test* | | **PD-non pRBD** | **PD-pRBD** | **HC** |
| --- | --- | --- | --- | --- |
| MMSE | | -0.46 (1.34) | -1.53 (3.17) | -0.39 (1.01) |
| Digit Span | |  |  |  |
|  | Forward | -0.24 (0.84) | -0.20 (0.88) | -0.11 (0.92) |
|  | Backward | -0.10 (0.78) | 0.01 (1.05) | 0.00 (0.72) |
|  | Forward minus Backward | -0.24 (1.29) | -0.31 (1.10) | -0.19 (0.82) |
| Phonetic fluency "p" | | -0.17 (1.24) | -0.62 (1.09) | -0.11 (1.22) |
| Semantic fluency "Animals" | | -0.78 (1.21) | -1.12 (1.43) | -0.10 (0.76) |
| Stroop | |  |  |  |
|  | Word | -0.48 (1.05) | -1.17 (1.40) | -0.04 (0.92) |
|  | Color | -0.30 (0.93) | -0.87 (1.09) | 0.24 (0.67) |
|  | Word-Color | 0.03 (0.94) | -0.57 (1.07) | 0.23 (0.72) |
| TMT | |  |  |  |
|  | A | -0.71 (2.09) | -2.70 (5.83) | 0.04 (0.64) |
|  | B | -1.29 (3.62) | -2.37 (5.42) | -0.02 (1.16) |
|  | B minus A | -3.52 (2.79) | - 4.54 (4.73) | -2.57 (0.96) |
| SDMT | | -0.46 (1.22) | -0.83 (1.51) | 0.00 (0.70) |
| RAVLT | |  |  |  |
|  | Total | 0.01 (1.51) | -0.73 (1.65) | 0.29 (0.90) |
|  | Recall | -0.36 (1.63) | -0.65 (1.54) | 0.18 (0.92) |
|  | True recognition | -0.26 (1.71) | -0.12 (1.07) | 0.31 (0.81) |
| JLO | | 0.17 (1.18) | -0.20 (1.42) | 0.46 (0.70) |
| BVFD | | 0.11 (0.86) | -0.39 (1.35) | 0.02 (0.77) |
| BFRT-short | | -0.19 (1.10) | -0.48 (1.21) | 0.45 (0.82) |
| BNT | | 0.10 (0.98) | 0.03 (0.81) | 0.18 (0.71) |

Neuropsychological performance descriptive statistics of PD-non pRBD and PD-pRBD on z-scores based on HC healthy control reference group^24^

Data are presented as mean (SD) of z-scores.

Abbreviations: PD-non pRBD = Parkinson’s disease patients without probable REM sleep behavior disorder; PD-pRBD = Parkinson’s disease patients with probable REM sleep behavior disorder; HC = healthy controls; MMSE = Mini-Mental State Examination; TMT = Trail Making Test; SDMT = Symbol Digit Modalities Test; RAVLT = Rey Auditory Verbal Learning Test; JLO = Benton Judgment of Line Orientation; BVFD = Benton Visual Form Discrimination; BFRT = Benton Facial Recognition Test; BNT = Boston Naming Test.

^24^ Cortical thinning associated with mild cognitive impairment in Parkinson’s disease. Mov. Disord. **29**, 1495-1503 (2014).

**Supplementary Table 4**

Connections with reduced functional connectivity in PD-pRBD patients compared with HC found by TFNBS

| **Cortico-cortical connections** | | | | |
| --- | --- | --- | --- | --- |
| **Node 1** | |  | **Node 2** | |
| **Label** | **Cytoarchitectonic correspondence*** |  | **Label** | **Cytoarchitectonic correspondence** |
| PCL_L_2_2 | A4ll, area 4, (lower limb region) | **—** | CG_L_7_6 | A23c, caudal area 24 |
| PCL_L_2_2 | A4ll, area 4, (lower limb region) | **—** | CG_R_7_6 | A23c, caudal area 24 |
| SFG_R_7_5 | A6m, medial area 6 | **—** | CG_L_7_4 | A23v, ventral area 23 |
| INS_R_6_2 | vIa, ventral agranular insula | **—** | CG_L_7_5 | A24cd, caudodorsal area 24 |
| INS_R_6_2 | vIa, ventral agranular insula | **—** | CG_L_7_6 | A23c, caudal area 24 |
| INS_R_6_2 | vIa, ventral agranular insula | **—** | CG_R_7_6 | A23c, caudal area 24 |
| STG_L_6_3 | Te1.0 and Te1.2 | **—** | CG_L_7_5 | A24cd, caudodorsal area 24 |
| STG_L_6_3 | Te1.0 and Te1.2 | **—** | CG_L_7_6 | A23c, caudal area 24 |
| STG_L_6_4 | A22c, caudal area 22 | **—** | CG_R_7_6 | A23c, caudal area 24 |
| STG_L_6_4 | A22c, caudal area 22 | **—** | IPL_R_6_4 | A40c, caudal area 40 (PFm) |
| **Cortico-DGM connections** | | | | |
| **Node 1** | |  | **Node 2** | |
| **Label** | **Cytoarchitectonic correspondence** |  | **Label** | **Cytoarchitectonic correspondence** |
| SFG_R_7_5 | A6m, medial area 6 | **—** | Str_L_6_2 | GP, globus pallidus |
| SFG_R_7_5 | A6m, medial area 6 | **—** | Str_L_6_6 | dlPu, dorsolateral putamen |
| CG_R_7_6 | A23c, caudal area 24 | **—** | Tha_L_8_8 | lPFtha, lateral pre-frontal thalamus |
| IPL_L_6_3 | A40rd, rostrodorsal area 40(PFt) | **—** | Str_L_6_4 | vmPu, ventromedial putamen |
| IPL_R_6_4 | A40c, caudal area 40(PFm) | **—** | Tha_L_8_8 | lPFtha, lateral pre-frontal thalamus |
| MTG_R_4_3 | A37dl, dorsolateral area 37 | **—** | Amyg_L_2_2 | lAmyg, lateral amygdala |

Significant reduced connections in Parkinson’s disease patients with probable REM sleep behavior disorder (PD-pRBD) compared with healthy controls (HC) found by threshold-free network-based statistics (TFNBS) with sex and maximum translation as covariates (*p* < 0.001, FWE corrected). Labels correspond with Brainnetome Atlas (see Supplementary Material 1 for more detail).

Abbreviations: CG = cingulate gyrus; INS = insular gyrus; IPL = inferior parietal lobule; L = left; MTG = middle temporal gyrus; PCL = paracentral lobule; R = right; SFG = superior frontal gyrus; STG = superior temporal gyrus; Str = striatum; Tha = thalamus.

*Based on Fan, L. *et al.* The Human Brainnetome Atlas: A New Brain Atlas Based on Connectional Architecture. *Cereb. Cortex* **26**, 3508–3526 (2016).

**Supplementary Table 5**

Summary of the sixteen significant reduced connections in PD-pRBD patients compared with HC found by TFNBS

| **Cortico-cortical connections** | | |  |  |
| --- | --- | --- | --- | --- |
|  | **Frontal-Limbic** | **Temporal-Parietal** | **Temporal-Limbic** | **Insular-Limbic** |
|  | 3 | 1 | 3 | 3 |
| **Cortico-Deep gray matter connections** | | |  |  |
|  | **Frontal-DGM** | **Temporal-DGM** | **Parietal-DGM** | **Limbic-DGM** |
|  | 2 | 1 | 2 | 1 |
|  | **LH-LH** | **RH-RH** | **LH-RH/RH-LH** |  |
|  | 4 | 1 | 11 |  |

Summary of the significant reduced connections in Parkinson’s disease patients with probable REM sleep behavior disorder (PD-pRBD) compared with healthy controls (HC) found by threshold-free network-based statistics (TFNBS) with sex and maximum translation as covariates (*p* < 0.001, FWE corrected).

Abbreviations: DGM = deep gray matter; LH = Left hemisphere; RH = Right hemisphere.

**Supplementary Figure 1.** Shows the normalized characteristic path length (nCPL) increment in PD-pRBD-MCI patients compared with PD-non pRBD-MCI. Normalized characteristic path length (vertical axis) as a function of sparsity thresholds (horizontal axis) for PD-pRBD-MCI and PD-non pRBD-MCI. (*) indicate significant differences between PD-non pRBD-MCI and PD-pRBD-MCI.

**
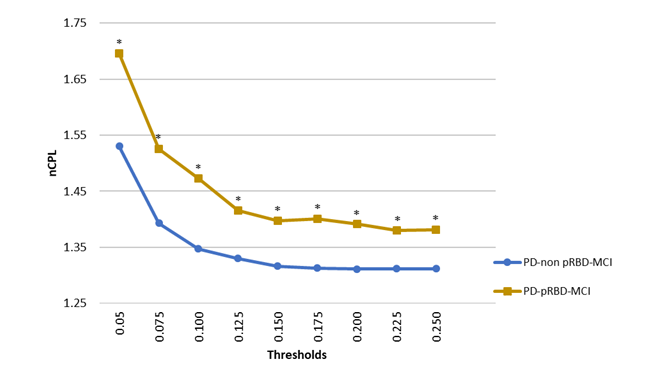
**

**Supplementary Methods 1**

Brainnetome atlas nodes and corresponding anatomical regions

| **Cingulate** |
| --- |
| CG_L(R)_7_1, CG_L(R)_7_2, CG_L(R)_7_3, CG_L(R)_7_4, CG_L(R)_7_5, CG_L(R)_7_6, CG_L(R)_7_7 |
| **Frontal** |
| SFG_L(R)_7_1, SFG_L(R)_7_2, SFG_L(R)_7_3, SFG_L(R)_7_4, SFG_L(R)_7_5, SFG_L(R)_7_6, SFG_L(R)_7_7, MFG_L(R)_7_1, MFG_L(R)_7_2, MFG_L(R)_7_3, MFG_L(R)_7_4, MFG_L(R)_7_5, MFG_L(R)_7_6, MFG_L(R)_7_7, IFG_L(R)_6_1, IFG_L(R)_6_2, IFG_L(R)_6_3, IFG_L(R)_6_4, IFG_L(R)_6_5, IFG_L(R)_6_6, OrG_L(R)_6_1, OrG_L(R)_6_2, OrG_L(R)_6_3, OrG_L(R)_6_4, OrG_L(R)_6_5, OrG_L(R)_6_6, PrG_L(R)_6_1, PrG_L(R)_6_2, PrG_L(R)_6_3, PrG_L(R)_6_4, PrG_L(R)_6_5, PrG_L(R)_6_6, PCL_L(R)_2_1, PCL_L(R)_2_2 |
| **Insula** |
| INS_L(R)_6_1, INS_L(R)_6_2, INS_L(R)_6_3, INS_L(R)_6_4, INS_L(R)_6_5, INS_L(R)_6_6 |
| **Medial temporal** |
| FuG_L(R)_3_1, FuG_L(R)_3_2, FuG_L(R)_3_3, PhG_L(R)_6_1, PhG_L(R)_6_2, PhG_L(R)_6_3, PhG_L(R)_6_4, PhG_L(R)_6_5, PhG_L(R)_6_6, Amyg_L(R)_2_1, Amyg_L(R)_2_2, Hipp_L(R)_2_1, Hipp_L(R)_2_2 |
| **Occipital** |
| MVOcC_L(R)_5_1, MVOcC_L(R)_5_2, MVOcC_L(R)_5_3, MVOcC_L(R)_5_4, MVOcC_L(R)_5_5, LOcC_L(R)_4_1, LOcC_L(R)_4_2, LOcC_L(R)_4_3, LOcC_L(R)_4_4, LOcC_L(R)_2_1, LOcC_L(R)_2_2 |
| **Parietal** |
| SPL_L(R)_5_1, SPL_L(R)_5_2, SPL_L(R)_5_3, SPL_L(R)_5_4, SPL_L(R)_5_5, IPL_L(R)_6_1, IPL_L(R)_6_2, IPL_L(R)_6_3, IPL_L(R)_6_4, IPL_L(R)_6_5, IPL_L(R)_6_6, Pcun_L(R)_4_1, Pcun_L(R)_4_2, Pcun_L(R)_4_3, Pcun_L(R)_4_4, PoG_L(R)_4_1, PoG_L(R)_4_2, PoG_L(R)_4_3, PoG_L(R)_4_4 |
| **Striatum** |
| STR_L(R)_6_1, STR_L(R)_6_2, STR_L(R)_6_3, STR_L(R)_6_4, STR_L(R)_6_5, STR_L(R)_6_6 |
| **Temporal** |
| STG_L(R)_6_1, STG_L(R)_6_2, STG_L(R)_6_3, STG_L(R)_6_4, STG_L(R)_6_5, STG_L(R)_6_6, MTG_L(R)_4_1, MTG_L(R)_4_2, MTG_L(R)_4_3, MTG_L(R)_4_4, ITG_L(R)_7_1, ITG_L(R)_7_2, ITG_L(R)_7_3, ITG_L(R)_7_4, ITG_L(R)_7_5, ITG_L(R)_7_6, ITG_L(R)_7_7, pSTS(R)_2_1, pSTS_L(R)_2_2 |
| **Thalamus** |
| Tha_L(R)_8_1, Tha_L(R)_8_2, Tha_L(R)_8_3, Tha_L(R)_8_4, Tha_L(R)_8_5, Tha_L(R)_8_6, Tha_L(R)_8_7, Tha_L(R)_8_8 |

This table collects all the nodes used in the functional connectivity and graph analyses and facilitates results interpretation. Further, the correspondence between label names with specific areas into the brain regions and corresponding MNI coordinates can be consulted on <http://cercor.oxfordjournals.org/content/26/8/3508.full.pdf> .

Abbreviations: CG = cingulate gyrus; SFG = superior frontal gyrus; MFG = middle frontal gyrus; IFG = inferior frontal gyrus; OrG = orbital gyrus; PrG = precentral gyrus; PCL = paracentral lobule; INS= insula; FuG = fusiform gyrus; PhG = parahippocampal gyrus; Amyg = amygdala; Hipp = hippocampus; MVOcC = medioventral occipital cortex; LOcC = lateral occipital cortex; SPL = superior parietal lobule; IPL = inferior parietal lobule; Pcun = precuneus; PoG = postcentral gyrus; Str = striatum; STG = superior temporal gyrus; MTG = middle temporal gyrus; ITG = inferior temporal gyrus; pSTS = posterior superior temporal sulcus; Tha = thalamus.
